# Retroperitoneal necrotizing fasciitis presenting with peritonism in a 33-year-old Nepalese man: a case report

**DOI:** 10.1186/1752-1947-6-53

**Published:** 2012-02-10

**Authors:** Smith Giri, Bishnu P Kandel, Prasan BS Kansakar, Pradeep Vaidya

**Affiliations:** 1 Department of Surgery, Tribhuvan University Teaching Hospital, Kathmandu, Nepal

## Abstract

**Introduction:**

Retroperitoneal necrotizing fasciitis is a rare, fulminant, and potentially lethal complication of intra-abdominal suppuration. A retroperitoneal origin is very rare and very few cases have been reported in the literature. To the best of our knowledge, this case is only the fourth case reported of successful management following retroperitoneal necrotizing fasciitis.

**Case presentation:**

A 33-year-old Tamang man presented to our facility with a history of five days of fever and vomiting and eight days of severe left loin pain. On examination, he had features of peritonism. A laparotomy was performed, revealing extensive necrotizing fasciitis of the retroperitoneum extending to the anterior abdominal wall. Our patient survived following extensive debridement of the necrotic tissues and supportive care.

**Conclusions:**

Retroperitoneal necrotizing fasciitis can rarely present with features of peritonism, and hence should be included as a possible differential diagnosis for anyone presenting with peritonism. Although a fatal condition, early intervention and aggressive management can save the life of a patient.

## Background

Necrotizing fasciitis (NF) is a relatively rare but rapidly spreading necrotizing infection of the subcutaneous tissues. It is caused by rapid proliferation of microorganisms. Originally described by Wilson in 1952, studies have reported varying mortality rates ranging from 20% to 40% [1]. Patients with compromised immune systems, such as those with diabetes mellitus, chronic renal failure, drug misuse, advanced age and other immunocompromised states, are at an increased risk of developing NF [1].

The diagnosis of NF is purely clinical, with laboratory parameters suggesting, but not confirming, the diagnosis [2]. It can occur in any region of the body but most commonly manifests in the extremities, abdominal wall and perineum [3]. Retroperitoneal necrotizing fasciitis is extremely rare [4], with very few cases reported in the literature. Here, we present the case of a 33-year-old man with retroperitoneal necrotizing fasciitis.

## Case presentation

A 33-year-old Tamang man presented to the Emergency Department of Tribhuvan University Teaching Hospital with the chief complaints of fever, multiple episodes of non-bilious vomiting over the last eight days and severe left loin pain for the last five days. He reported trauma to his back about three weeks ago associated with backache, subsiding in a few days. There was no other significant history. Our patient chewed tobacco regularly and occasionally consumed alcohol.

On examination, he had a pulse rate of 120 beats/minute and temperature of 38.3°C. Abdominal examination revealed generalized rigidity and guarding along with other features of peritonism. His renal angles were non-tender. There were no other findings of significance.

Investigations revealed a raised white blood cell count (WBC; 24,900 cells/mm^3^). Blood sugar level, urea, creatinine and urine microscopic examination and culture results were all normal. Serum amylase and lipase levels, along with the results of a chest X-ray were normal. An abdominal ultrasound scan revealed a minimal amount of free fluid in the pelvis. Aspiration revealed a purulent exudative fluid (total count of 25 000 cells per mm^3^).

**Figure 1:**
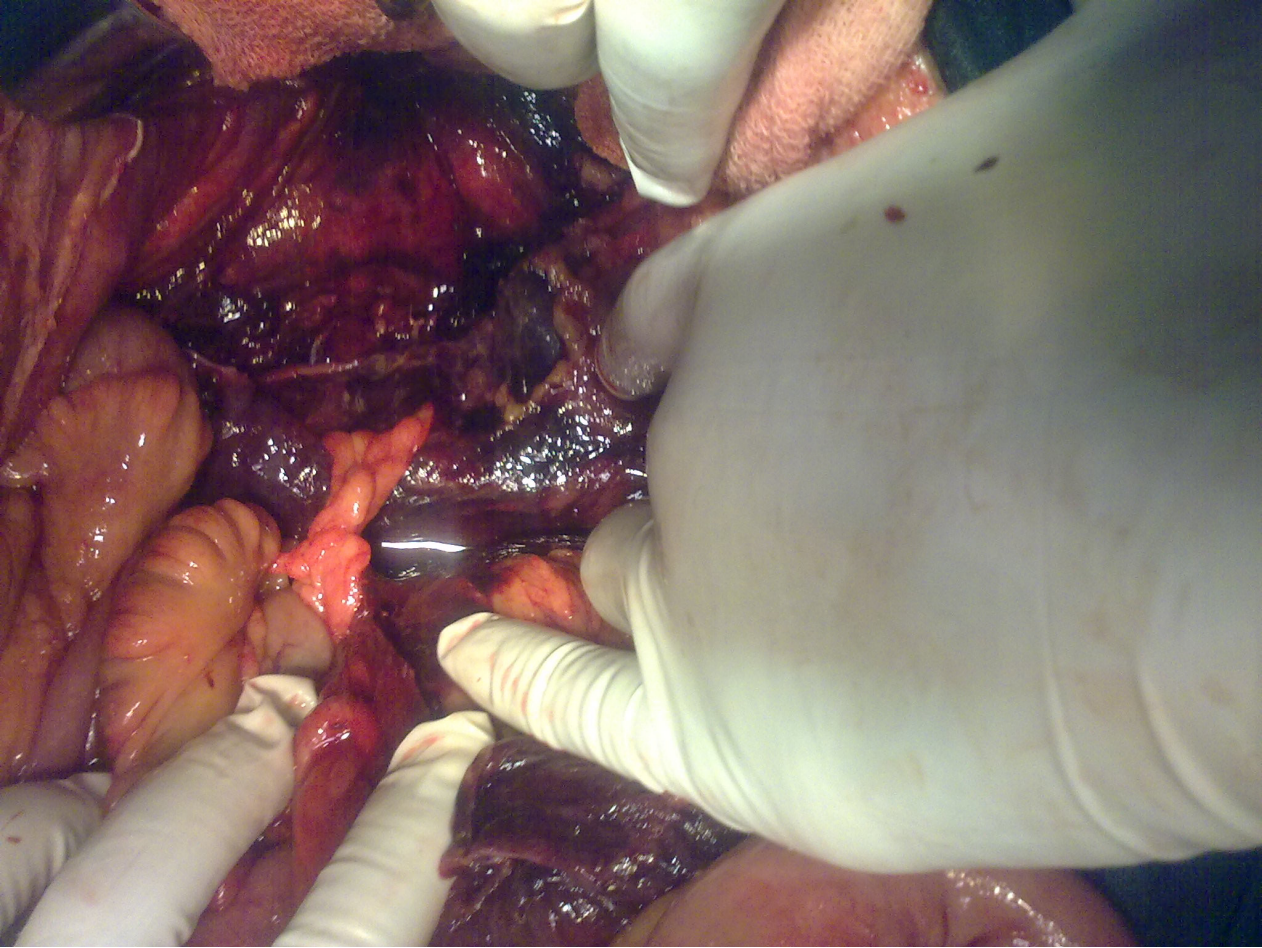
**Peri-operative findings in our patient**. Peri-operative findings revealed minimal purulent fluid in the peritoneal cavity, with extensive necrosis of the retroperitoneum spreading to the anterior abdominal wall. The intestine and solid organs including the kidneys were normal.

With a provisional diagnosis of enteric perforation peritonitis, our patient was immediately taken to the operation theatre for laparotomy. Peri-operative findings showed minimal purulent fluid in the peritoneal cavity. There was extensive necrosis of the retroperitoneum spreading to the left side of the anterior abdominal wall (parietal peritoneum) (Figure 1). A differential diagnosis of necrotizing fasciitis was not initially suspected, thus a laboratory risk indicator for necrotizing fasciitis (LRINEC) score was not calculated pre-operatively. An incidental diagnosis of retroperitoneal necrotizing fasciitis was made under observation during laparotomy. Colonic perforation and peri-renal abscess were excluded with findings of normal intestines and solid visceral organs including kidneys. Pyelonephritis was excluded based on the results of urine microbiological examination and culture.

Debridement of the necrotic tissue was performed with placement of a corrugated retroperitoneal drain and a pelvic tube drain. Suspecting infection due to pathogens originating from the gastrointestinal tract, we started our patient on intravenous cefoperazone, sulbactam and clindamycin to cover anaerobic and Gram-negative enterobacteria. Necrotic tissue and drainage fluid culture revealed *Escherichia coli *sensitive to cefoperazone. The same antibiotics were continued post-operatively. Our patient improved with no post-operative complications and was discharged on the 12th post-operative day.

## Discussion

NF is a serious soft tissue infection that causes secondary necrosis of the soft tissues. We report the case of a patient who developed extensive necrotizing fasciitis of the retroperitoneum. The diagnosis was confirmed intra-operatively with subsequent debridement, and he was treated successfully without complications. Of the reported cases of retroperitoneal necrotizing fasciitis in the literature, most had identifiable sources of infection including chronic pyelonephritis, diverticulitis, peri-anal abscess, colonic cancer, perforation, urinary extravasation and post-hemorrhoidectomy [2,3,5-7]. As with our patient, there have been cases of retroperitoneal necrotizing fasciitis reported without an obvious source of infection [8-10]. Our patient revealed a history of local trauma three weeks prior, followed by mild loin pain over a few days, that was tolerated by our patient until it subsided. We hypothesize that the patient developed a retroperitoneal hematoma following the traumatic event, which subsequently developed an infection. While retroperitoneal necrotizing fasciitis is more common in immunocompromised patients [7], our patient had no history or clinical findings suggestive of diabetes mellitus, chronic renal failure, human immunodeficiency virus or any other obvious immunosuppressed state.

Clinically, necrotizing fasciitis presents with a wide range of signs and symptoms, therefore early diagnosis and treatment is a major challenge. Previously reported cases of retroperitoneal necrotizing fasciitis have presented with various clinical findings including fever and abdominal pain [7], features of peritonitis [8,9], flank pain [6], features mimicking appendicitis [10] as well as fever with abdominal pain, skin erythema and crepitus [5]. Our patient's presentation of fever and features of peritonitis is hence not in isolation [8,9].

Similar cases of retroperitoneal NF presenting with features of peritonitis have been reported by Jayatunga *et al. *in 1993 and Sugimoto *et al. *in 2010 [8,9]. In the former, the patient was a 74-year-old diabetic woman with retroperitoneal necrotizing fasciitis limited to her pelvis [8]. In the latter, the patient was a 58-year-old hypertensive man with no obvious immunocompromised state who had developed extensive necrotizing fasciitis of the retroperitoneum. He also developed Fournier's gangrene post-operatively. A post-operative colonoscopy revealed a colonic adenocarcinoma [9]. In the case reported by Jayatunga *et al*., microbiology revealed *E. coli *and *Streptococcus faecalis *sensitive to vancomycin and ceftazidime [8]. But in the case reported by Sugimoto *et al*., *Streptococcus anginosus *was isolated [9]. In our case *E. coli *was isolated that was sensitive to cefoperazone, however, we continued to cover for anaerobic bacteria as well. As in our patient's case, both these cases had an unclear etiology and both patients survived following extensive debridement of the necrotic tissues and supportive care.

Retroperitoneal necrotizing fasciitis is associated with a high mortality rate, with survival reported in very few cases [8-10]. Several factors could have helped contribute to the positive outcome for our patient. These include our patient's age and absence of any comorbidities, urgent laparotomy being undertaken, and the extensive debridement of necrotizing tissues. Appropriate antibiotic treatment, good nursing care and proper metabolic and nutritional support would have contributed to a complication-free post-operative period.

## Conclusions

We report a case of retroperitoneal necrotizing fasciitis with an unclear etiology. Although severe and extensive inflammatory necrosis was found throughout the retroperitoneum, our patient survived due to rapid and aggressive surgical intervention, broad-spectrum antibiotics and good supportive care.

## Consent

Written informed consent was obtained from the patient for publication of this case report and any accompanying images. A copy of the written consent is available for review by the Editor-in-Chief of this journal.

## Competing interests

The authors declare that they have no competing interests.

## Authors' contributions

SG wrote the manuscript. SG, BPK and PBSK gathered the clinical information, took consent from our patient and performed the literature review. PV contributed to the final manuscript and critically reviewed the intellectual content of the manuscript. All authors read and approved the final manuscript.
